# Efficacy and safety of concurrent immune checkpoint inhibitors combined with radiotherapy or chemoradiotherapy for advanced non-small cell lung cancer: A systematic review and single-arm meta-analysis

**DOI:** 10.1371/journal.pone.0304941

**Published:** 2024-06-12

**Authors:** Ran Cui, Yun Li, Xinlin Yu, Chun Wei, Ou Jiang

**Affiliations:** 1 Department of Oncology, The First People’s Hospital of Neijiang, Neijiang, Sichuan, China; 2 Department of Oncology, The Second People’s Hospital of Neijiang, Neijiang, Sichuan, China; Chung Shan Medical University, TAIWAN

## Abstract

**Background:**

The recent usage of immunotherapy combined with chemoradiotherapy has improved survival in advanced non-small cell lung cancer (NSCLC) patients. However, determining the most effective therapy combination remains a topic of debate. Research suggests immune checkpoint inhibitors (ICIs) post-chemoradiotherapy enhance survival, but the impact of concurrent ICIs during chemoradiotherapy on rapid disease progression is unclear. This meta-analysis aims to assess the effectiveness and safety of concurrent ICIs with radiotherapy or chemoradiotherapy in advanced non-small cell lung cancer.

**Methods:**

We searched PubMed, Embase, the Cochrane Library, and Web of Science for relevant studies, extracting data on overall response rate (ORR), progression-free survival (PFS), overall survival (OS), and adverse events (AEs).

**Results:**

The analysis included ten studies with 490 participants. Stage III NSCLC ORR was 81.8%, while Stage IV ORR was 39.9%. One-year PFS and OS for Stage III were 68.2% and 82.6%, compared to 27.9% and 72.2% for Stage IV. Common adverse events included anemia (46.6%), nausea (47.6%), rash (36.4%), and radiation pneumonitis (36.3%).

**Conclusions:**

Our meta-analysis shows concurrent ICIs with chemoradiotherapy are effective and safe in advanced NSCLC, particularly in stage III patients at risk of progression before starting ICIs after chemoradiotherapy. The findings support further phase III trials. The review protocol was registered on PROSPERO (CRD42023493685) and is detailed on the NIHR HTA programme website.

## Introduction

Lung cancer constitutes the primary cause of cancer-related mortality globally [1]. Non-small cell lung cancer (NSCLC) accounts for roughly 80% to 85% of all lung cancer cases, with advanced NSCLC comprising over 20% of cases at initial diagnosis [2, 3]. In patients with inoperable and advanced NSCLC, definitive concurrent chemoradiation therapy (cCRT) traditionally represented the standard of care (SoC). However, the long-term results of cCRT were unsatisfactory, exhibiting a 5-year survival rate of only 15% to 30% [4]. ICIs for NSCLC, particularly advanced NSCLC, have emerged as an effective treatment approach, as numerous clinical trials have substantiated their clinical advantages [5–7]. The integration of ICIs with radiotherapy or chemotherapy has been vigorously pursued, consistently yielding positive outcomes since the pivotal findings of the PACIFIC trial in 2017 [8–11]. Presently, the consolidative use of ICIs is globally sanctioned by health authorities and embraced in guidelines as the SoC [12].

The role of radiation therapy in modulating the immune system, thereby creating an environment conducive to anti-tumor immunity, is increasingly recognized [13, 14]. Radiation therapy is noted for its diverse immunomodulatory effects, including enhanced antigen presentation, chemokine secretion, effector T-cell recruitment to the tumor site, and promoting immunogenic cell death driven by lymphocytes [15]. Additionally, post-radiation activation of inhibitory T-cell regulatory pathways, such as the PD-1/PD-L1 axis, has been observed, contributing to these immunological results [16]. Importantly, radiation therapy can induce an increase in PD-1 and PD-L1 expression on both immune and tumor cells, highlighting the strategic value of combining it with PD-1/PD-L1 inhibitors [17]. However, recent evidence indicates that the treatment schedule, particularly the timing and sequence of radiotherapy–immunotherapy combinations, is crucial for efficacy. The ideal timing for integrating ICIs with radiotherapy or chemoradiotherapy to achieve synergistic effects requires additional research [18]. The PACIFIC trial recommended initiating durvalumab within 1 to 42 days after radiotherapy. In contrast, current NCCN guidelines recommend starting durvalumab after completing both radiotherapy and chemotherapy, without specifying an exact start time following radiotherapy. Furthermore, recent studies have explored the feasibility of concurrent ICIs with radiotherapy or chemoradiotherapy, reporting outcomes that suggest significant therapeutic potential. The simultaneous use of ICIs with radiotherapy or chemoradiotherapy has shown promising results, underscoring the need for more in-depth examination of this combined approach. The concurrent therapeutic strategies involving ICIs and radiotherapy not only demand further investigation but also refinement to optimize clinical effectiveness.

Moreover, the adverse events associated with combining ICIs with radiotherapy or chemoradiotherapy are a concern, as balancing efficacy and safety is vital. The concurrent use of ICIs with radiotherapy or chemoradiotherapy may lead to systemic effects, particularly an increased risk of immune-related pneumonitis, the most severe of all reported adverse outcomes. This meta-analysis is designed to evaluate the efficacy and safety of combining ICIs with radiotherapy or chemoradiotherapy in treating advanced non-small cell lung cancer. The expected findings of this study could expand the spectrum of clinical management options available.

## Materials and methods

### Search strategy

Four databases–PubMed, Embase, the Cochrane Library, and Web of Science–were thoroughly searched for pertinent studies. The final search date was 4 January 2024. The search strategy incorporated both MeSH terms and free-text words: “concurrent radiotherapy” OR “concurrent radiation therapy” OR “concurrent chemoradiotherapy” OR “concurrent radiotherapy AND immunotherapy” OR “concurrent chemoradiotherapy AND immunotherapy” AND (“immune checkpoint inhibitors” OR “PD-1 inhibitors” OR “PD-L1 inhibitors” OR “CTLA-4 inhibitors” OR “immune modulation” OR “immunotherapy”) AND “advanced NSCLC” OR “advanced non-small cell lung cancer”. Searches were restricted to English language publications. Additionally, the references of the included articles were reviewed to identify further relevant studies.

### Selection criteria

Studies were included in this meta-analysis if they met the following inclusion criteria: 1) population: patients diagnosed with advanced non-small cell lung cancer (NSCLC); and 2) intervention: patients treated with concurrent immune checkpoint inhibitors (ICIs) combined with radiotherapy/chemoradiotherapy. Study Type: Prospective interventional research, retrospective analyses, or randomized controlled trials (RCTs). 3) Outcomes: Clinical tumor outcomes of interest, including objective response rate (ORR), one-year progression-free survival (PFS), one-year overall survival (OS), and adverse events (AEs), were reported. 4) Tumor responses were evaluated using the Response Evaluation Criteria in Solid Tumors (RECIST) [19], version 1.1. Toxic effects were assessed for incidence and severity using the Common Terminology Criteria for Adverse Events (CTCAE). The exclusion criteria were as follows: Animal-related studies, cell studies, reviews, meta-analyses, duplicates, case reports, or letters.

Two investigators independently screened the articles for eligibility using the inclusion and exclusion criteria. Any disagreements regarding study selection were resolved by discussion between the two investigators or with the involvement of a third investigator.

### Data extraction and quality assessment

Two investigators independently extracted data from all included studies and also performed a quality assessment of the studies. The extracted data included author name, publication year, study type, sample size, intervention, tumor stage, median follow-up time, EGFR mutation status, and reported endpoints. Clinical and safety outcomes were evaluated based on the overall response rate (ORR), one-year overall survival (OS), one-year progression-free survival (PFS), incidence of any adverse events (AEs), and incidence of grade 3 or higher AEs.

Furthermore, the quality of the included randomized controlled trials (RCTs) was assessed using the Jadad scale, while the retrospective studies were evaluated using the Joanna Briggs Institute Critical Appraisal Checklist for Patient Series. The Newcastle–Ottawa Scale (NOS) was used to evaluate the quality of the included noncontrolled trials.

### Statistical analysis

This meta-analysis was conducted using STATA 17 software (StataCorp LP, College Station, TX, United States) to analyze the data. Heterogeneity among studies was assessed with the chi-square test and I^2^ statistic, with p values less than 0.1 denoting significant differences. In cases where there was significant variability (p < 0.1 and I^2^ > 50%), the analysis utilized a random effects approach. On the other hand, for scenarios with lower variability, a fixed-effects approach was chosen. Furthermore, sensitivity analyses were performed to evaluate the robustness and reliability of the findings. The possibility of publication bias was assessed using Begg’s and Egger’s tests.

### Ethics approval and consent to participate

This meta-analysis was conducted in accordance with the Declaration of Helsinki. Informed consent was obtained from the participants of all included studies, and the study was approved by their respective institutional ethics committees. As this study is a meta-analysis of previously published data, no additional informed consent was needed.

## Results

### Study selection

**Fig 1:**
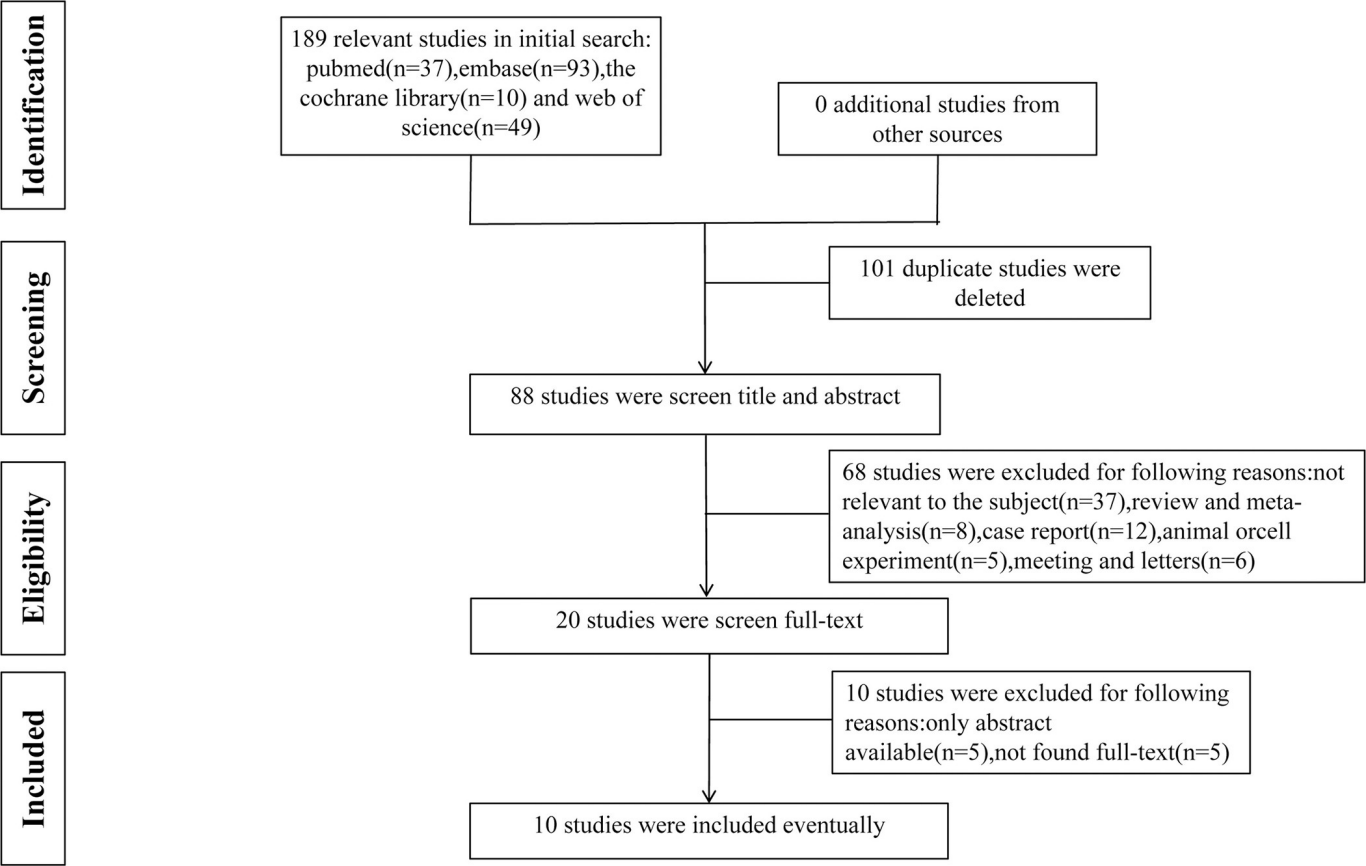
Flow diagram of the meta-analysis for the inclusion/exclusion of studies.

The initial search across four databases—PubMed (n = 37), Embase (n = 93), Cochrane Library (n = 10), and Web of Science (n = 49)—resulted in 189 relevant published studies. After removing duplicate articles and reviewing titles and abstracts, 20 studies remained. Further scrutiny of the full-text articles led to the exclusion of 10 studies due to the lack of available full text, insufficient sample sizes, or the investigation of nonchemotherapeutic drugs. Ultimately, ten studies, including 490 patients satisfied the inclusion criteria and were included in this meta-analysis. The selection process is depicted in Fig 1, and the specifics of each study are detailed in Table 1. The median age of participants ranged from 62 to 72 years across the studies. Male patients (n = 288) outnumbered female patients (n = 202). Adenocarcinoma (n = 238) and squamous cell carcinoma (n = 149) were the predominant histological types. Various radiotherapy techniques were employed, including 3D-CRT, VMAT, SBRT, SCRT, and proton therapy, with total radiation doses ranging from 45Gy to 66Gy. PD-L1 expression levels were reported in 7 studies, with more patients having TPS≥1% (n = 133) than TPS<1% (n = 69). Chemotherapy regimens mainly consisted of platinum-based drugs such as cisplatin or carboplatin combined with paclitaxel, pemetrexed, etoposide, or vinorelbine. The immune checkpoint inhibitors used included durvalumab, pembrolizumab, nivolumab, ipilimumab, sintilimab, and gefitinib. In most studies, ICIs were administered concurrently with chemoradiotherapy, starting on the same day. Further details of each study, including patient characteristics, treatment modalities, and sequences, are provided in Table 2.

**Table 1:** Characteristics of the studies included in the meta-analysis.

Study	Year	Study type	Sample size	Median follow-up (months)	Intervention	Tumor Stage	EGFR Mutation	Endpoints
Abe et al. [20]	2023	Prospective	27	22	Concurrent durvalumab with chemoradiotherapy	Stage IIIB/IIIC	N/A	ORR,1-year PFS 1-yearOS, AEs
Akamatsu et al. [21]	2021	Single-arm	27	51.8	Concurrent gefitinib and radiotherapy	Sage IIIA/IIIB	27	ORR, PFS OS, AEs
Bestvina et al. [22]	2022	RCT	18	17	Concurrent ipilimumab, nivolumab and radiotherapy	Stage IV	19	ORR, DCR, PFS 1-year OS, AEs
Liu et al. [23]	2022	Single-arm	30	41.7	Concurrent atezolizumab and chemoradiotherapy	Stage IV	4	ORR, PFS 1-year OS, AEs
Welsh et al. [24]	2020	RCT	80	20.4	Concurrent pembrolizumab and radiotherapy	Stage IIIB/IIIC	11	ORR, PFS 1-year OS, AEs
Tachihara et al. [25]	2023	Single-arm	35	22.8	Concurrent durvalumab with chemoradiotherapy	StageIIIB/IIIC	N/A	ORR,1-year PFS 1-year OS, AEs
Jabbour et al. [26]	2020	Single-arm	21	16	Concurrent pembrolizumab with chemoradiotherapy	Stage IIIA/IIIB	N/A	ORR, DCR, PFS, OS, AEs
Peters et al. [27]	2021	Single-arm	79	21	Concurrent nivolumab with chemoradiotherapy	Stage IIIA/IIIB	N/A	ORR, DCR, PFS, OS, AEs
Tang et al. [28]	2023	Prospective	49	22.7	Concurrent sintilimab with chemoradiotherapy	Stage IIIA/IIIB/IIIC	N/A	ORR,1-year PFS 1-year OS, AEs
Jabbour et al. [29]	2021	RCT	102	18.5	Concurrent pembrolizumab with chemoradiotherapy	Stage IIIA/IIIB/IIIC	N/A	ORR, DCR, 1-year PFS 1-year OS, AEs

**Table 2:** Raw data of the included studies.

Study	Abe et al. [20]	Akamatsu et al. [21]	Bestvina et al. [22]	Liu et al. [23]	Welsh et al. [24]	Tachihara et al. [25]	Jabbour et al. [26]	Peters et al. [27]	Tang et al. [28]	Jabbour et al. [29]
Age, years, median (range)	70 (62–79)	67 (45–74)	63.2, (45–78)	68 (50–83)	68 (52–81)	72 (44–83)	69.5 (53.0–85.0)	62.3 (60.3–64.3)	62 (45–75)	64.0 (35–81)
Sex, n										
Male	8	7	9	18	51	4	10	53	41	40
Female	4	20	9	12	29	31	11	26	8	62
Smoking status, n (%)										
Never-smoker	N/A	15	4	9	19	1	1	3	12	5
Smoker or ex-smoker	N/A	12	14	21	61	34	20	76	37	97
Histological type, n										
Adenocarcinoma	7	27	15	20	61	19	11	47	11	
Squamous cell carcinoma	4	0	2	7	17	15	10	28	38	
Non-small cell lung cancer	1	0	1	3	2	1	0	4	0	102
Radiotherapy technique, n	3D-CRT (n = 4), VMAT (n = 8)	3D-CRT(n = 27)	SBRT(n = 18)	SCRT(n = 24) Proton therapy(n = 6)	3D-CRT (n = 40), SBRT (n = 40)	3D-CRT (n = 24), SCRT (n = 11)	SCRT (n = 13) VMAT(n = 5)Proton therapy(n = 3)	3D-CRT (n = 79)	SCRT(n = 49)	3D-CRT (n = 102)
Total dose of radiotherapy	60Gy	64Gy	50Gy	66Gy	45Gy in 3D-CRT/50Gy SBRT	60Gy	60Gy	66Gy	60Gy	60Gy
ECOG performance status score, No. (%) a										
0	N/A	19	11	N/A	N/A	19	N/A	37	20	57
1	N/A	8	7	N/A	N/A	16	N/A	41	29	45
>2	N/A	0	0	N/A	N/A	0	N/A	1	0	0
PD-L1 status, n										
TPS<1%	4	N/A	6	8	19	N/A	4	N/A	N/A	28
TPS≥1%	7	N/A	12	17	32	N/A	15	N/A	N/A	40
Unevaluated	1	N/A	0	5	29	N/A	2	N/A	N/A	34
Chemotherapy treatment	carboplatin+paclitaxel, daily carboplatin, or cisplatin	N/A	N/A	N/A	N/A	Cisplatin + vinorelbine	Carboplatin + paclitaxel	Cisplatin + etoposide/pemetrexed/vinorelbine OR carboplatin + etoposide/pemetrexed/vinorelbine	Cisplatin + pemetrexed (nonsquamous) or cisplatin + etoposide (squamous)	Carboplatin + paclitaxel
immune checkpoint inhibitors treatment	Durvalumab 10mg/kg Q2W	Gefitinib 250 mg Daily	Nivolumab (3 mg/kg every 2 wk) + ipilimumab (1 mg/kg every 6 wk)	Durvalumab 10mg/kg Q2W	Pembrolizumab 200 mg every 3 weeks	Durvalumab 10mg/kg Q2W	Pembrolizumab 200mg Q3W	Nivolumab 360mg Q3W	Sintilimab 200mg Q3W	Pembrolizumab 200mg Q3W
Treatment sequence	N/A	ICIs was started on the same day as chemoradiation	received ICIs first and completed SBRT	ICIs was started on the same day as chemoradiation	ICIs was started on the same day as chemoradiation	ICIs was started on the same day as chemoradiation	ICIs was started on the same day as chemoradiation	ICIs was started on the same day as chemoradiation	ICIs was started on the same day as chemoradiation	ICIs was started on the same day as chemoradiation

### Quality assessment

The evaluation of the six non-randomized studies was conducted using the Newcastle-Ottawa Scale (NOS), which analyzes studies across three domains: selection of the study groups, comparability of the groups, and ascertainment of the outcome for cohort studies or the exposure for case-control studies, through eight specific criteria. Two retrospective studies were assessed using the Joanna Briggs Institute Critical Appraisal Checklist for Case Series, focusing on methodological quality across ten areas, including case selection, description of the condition or health issue, and clarity in presenting case details. The three included Randomized Controlled Trials (RCTs) were appraised using the Jadad Scale, which concentrates on three essential elements: the description of randomization, blinding, and accounting for lost-to-follow-up participants. Details of these quality assessments are provided in Table 3.

**Table 3:** Quality assessment of the studies included in the meta-analysis.

Newcastle–Ottawa Scale (NOS) for non-randomized studies											
Study	Q1	Q2	Q3	Q4	Q5	Q6	Q7	Q8			TOTAL
Akamatsu et al. [21]	1	0	1	1	0	1	0	1			5
Liu et al. [23]	1	0	1	1	0	1	0	1			5
Tachihara et al. [25]	1	0	1	1	0	1	0	1			5
Jabbour et al. [26]	1	0	1	1	0	1	0	1			5
Peters et al. [27]	1	0	1	1	0	1	0	1			5
Jabbour et al. [26]	1	0	1	1	0	1	0	1			5
Modified JADAD Scale for Reporting Randomized Controlled Trials											
Study	Q1	Q2	Q3	Q4							TOTAL
Bestvina et al. [22]	2	2	2	1							7
Welsh et al. [24]	2	2	2	1							7
Tang et al. [28]	2	2	2	1							7
JBI Critical Appraisal Checklist for Case Series for included retrospective studies											
Study	Q1	Q2	Q3	Q4	Q5	Q6	Q7	Q8	Q9	Q10	TOTAL
Abe et al. [20]	2	0	2	2	2	0	2	2	2	2	16

### Newcastle–Ottawa Scale (NOS) for non-randomized studies

The numbers Q1-Q18 in the heading signs were as follows: Ⅰ, representative of the exposed cohort; Ⅱ, representative of the nonexposed cohort; Ⅲ, representative of the exposed cohort; Ⅳ, representative of the outcome of interest was present at the start of the study; Ⅴ, representative of the cohorts on the basis of the design or analysis; Ⅵ, representative of the cohort assessment; and Ⅶ, long enough for outcomes to occur. VIII, adequacy of follow-up of cohorts.

### JADAD scale for reporting randomized controlled trials

Numbers Q1-Q4 in heading signified: Q1: Was the study described as randomized? Q2: Was the method of randomization appropriate (e.g., computer-generated random numbers)? Q3: Was the study described as double-blind? Q4: Was there a description of withdrawals and dropouts?

### JBI critical appraisal checklist for patient series for included retrospective studies

Numbers Q1-Q10 indicated the following inquiries: Q1, were criteria for inclusion in the case series clearly defined? Q2, was the condition assessed in a consistent, reliable manner for all participants in the case series? Q3, were reliable methods utilized for identifying the condition in all case series participants? Q4, did the case series include participants consecutively? Q5, was participant inclusion in the case series complete? Q6, were participant demographics in the study reported with clarity? Q7, was clinical information of the participants clearly reported? Q8, were case outcomes or follow-up findings clearly documented? Q9, was demographic information of the presenting site(s)/clinic(s) clearly documented? Q10, was the statistical analysis conducted appropriately?

### Tumor response

**Fig 2:**
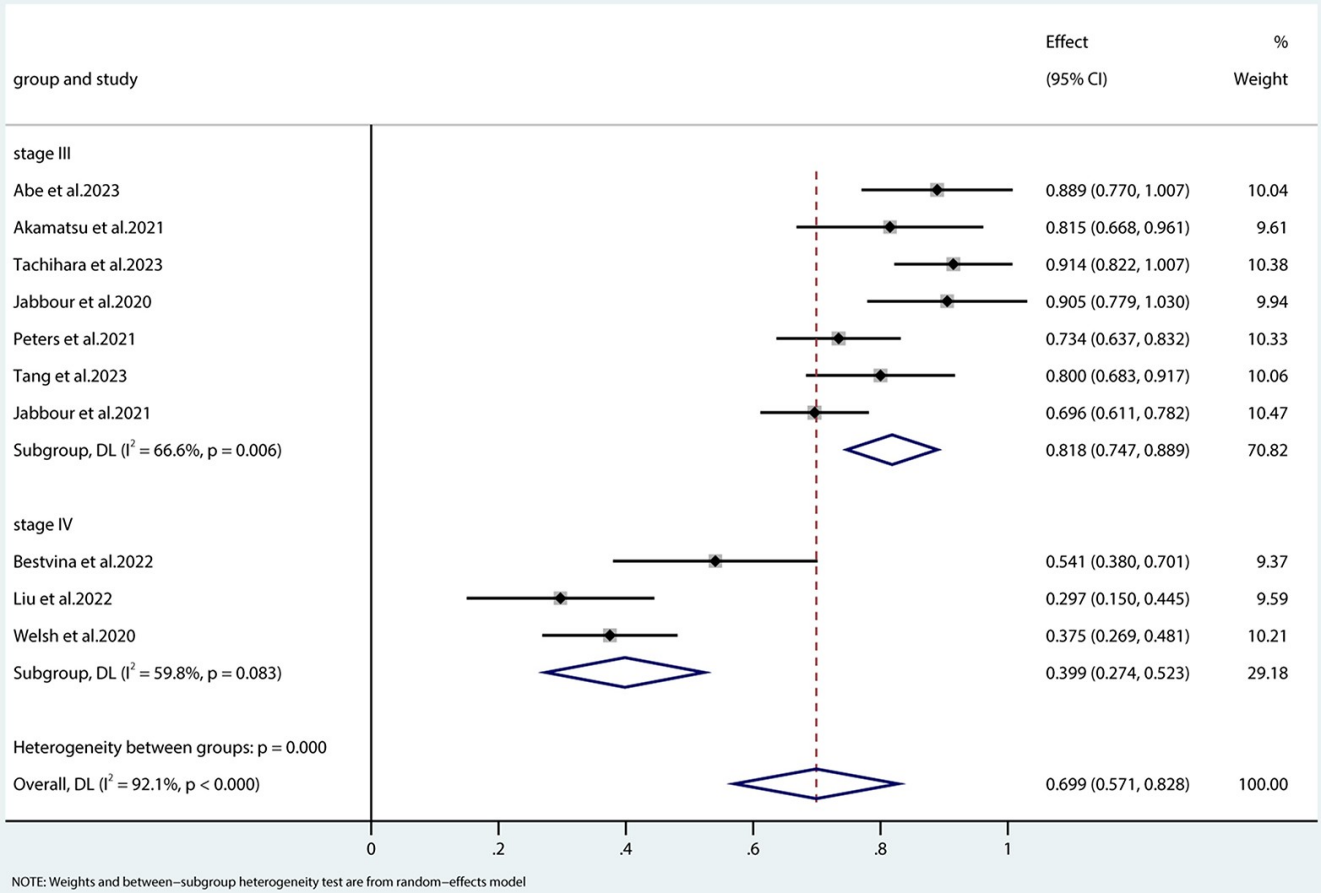
Forest plot of the pooled ORR. ORR, overall response rate; DCR, disease control rate.

All studies included in this analysis evaluated the effectiveness of concurrent immune checkpoint inhibitors (ICIs) combined with radiotherapy or chemoradiotherapy in treating advanced non-small cell lung cancer (NSCLC). The overall response rates (ORRs) observed in these studies varied widely, with a range from 30% to 90.9%. Owing to significant heterogeneity among the studies (I^2^ = 92.1%, p < 0.01), a random-effects model was utilized for the meta-analysis. The analysis revealed a combined ORR of 69.9% (95% CI: 57.1%–82.8%). When further stratifying the results by disease stage, it was found that for patients with stage III NSCLC treated with ICIs alongside radiotherapy, the ORR was 81.8% (95% CI: 74.7%–88.9%). Conversely, for patients with stage IV NSCLC, the ORR was 39.9% (95% CI: 27.4%–52.3%). These outcomes are graphically represented in Fig 2. Heterogeneity analysis indicated that the primary data originated from the study conducted by Jabbour et al.

### Survival

**Fig 3:**
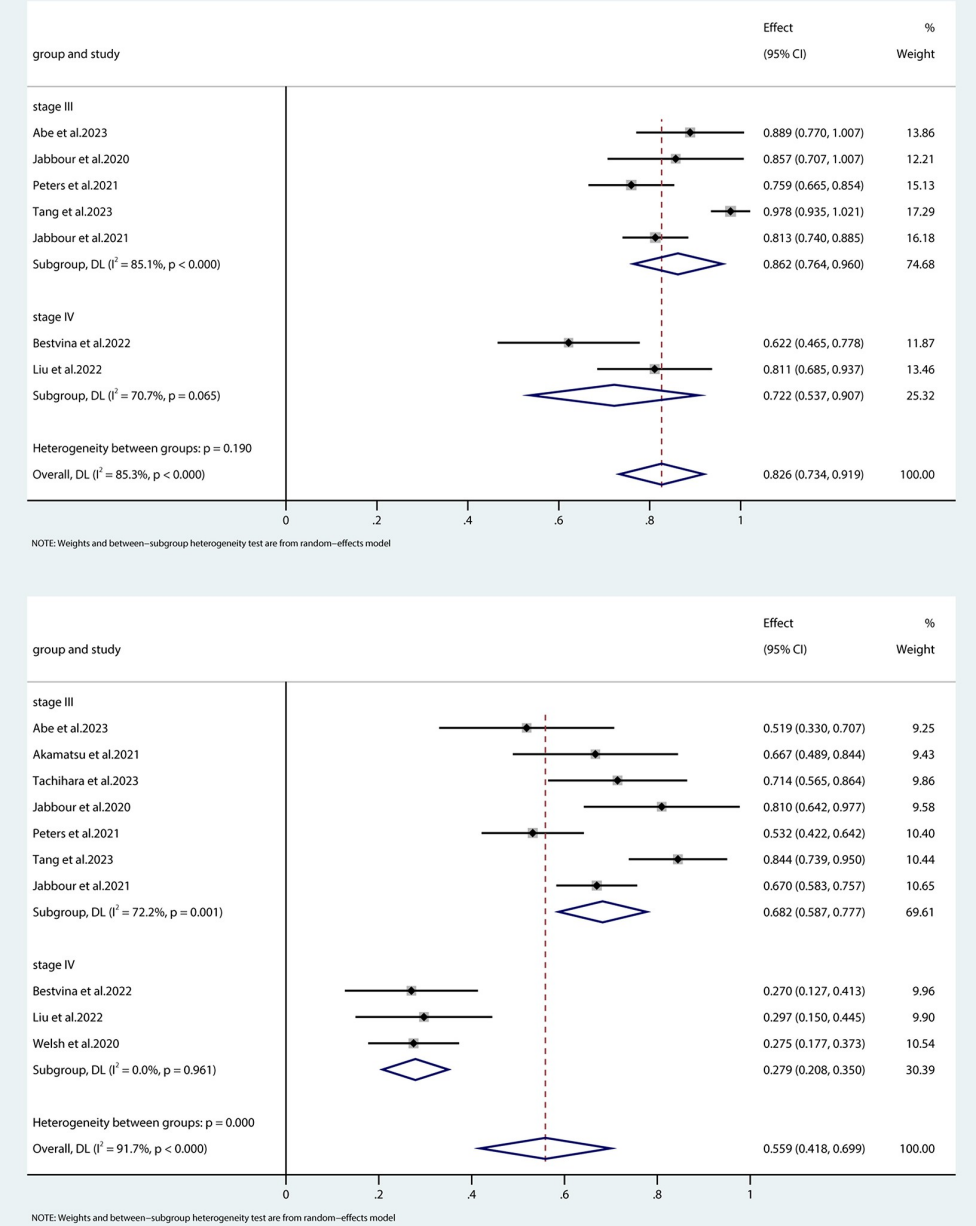
Forest plot of the pooled results for OS (A) and PFS (B) according to treatment regimen. OS, overall survival; PFS, progression-free survival.

Although some studies in the meta-analysis did not meet the predetermined endpoint, all provided data on one-year overall survival (OS) and progression-free survival (PFS) for patients receiving concurrent treatment with immune checkpoint inhibitors (ICIs) and radiotherapy or chemoradiotherapy. Employing a random-effects model (I^2^ = 85.3%, p < 0.001), the analysis generated a pooled one-year PFS rate of 55.9% (95% CI: 41.8%–69.9%). Upon stratification by disease stage, the one-year PFS was 68.2% (95% CI: 58.7%–77.7%) for patients with stage III non-small cell lung cancer (NSCLC), while for stage IV patients, the rate was 27.9% (95% CI: 20.8%–35.0%), as depicted in Fig 3A. The heterogeneity analysis indicates that the primary variability in data stems from the study conducted by Tang et al. Moreover, the one-year OS rate according to the random effects model was 82.6% (95% CI: 73.4%–91.9%), with stage III NSCLC patients demonstrating an OS rate of 86.2% (95% CI: 76.4%–96.0%) and stage IV patients showing a rate of 72.2% (95% CI: 53.7%–90.7%), as presented in Fig 3B.

### Subgroup analysis comparing different radiotherapy modalities

In this subgroup analysis, we explored how various radiotherapy modalities affect important efficacy measures, such as overall response rate (ORR), progression-free survival (PFS), and overall survival (OS) in patients with advanced non-small cell lung cancer (NSCLC) who received a combination of radiotherapy and immune checkpoint inhibitors.

**Fig 4:**
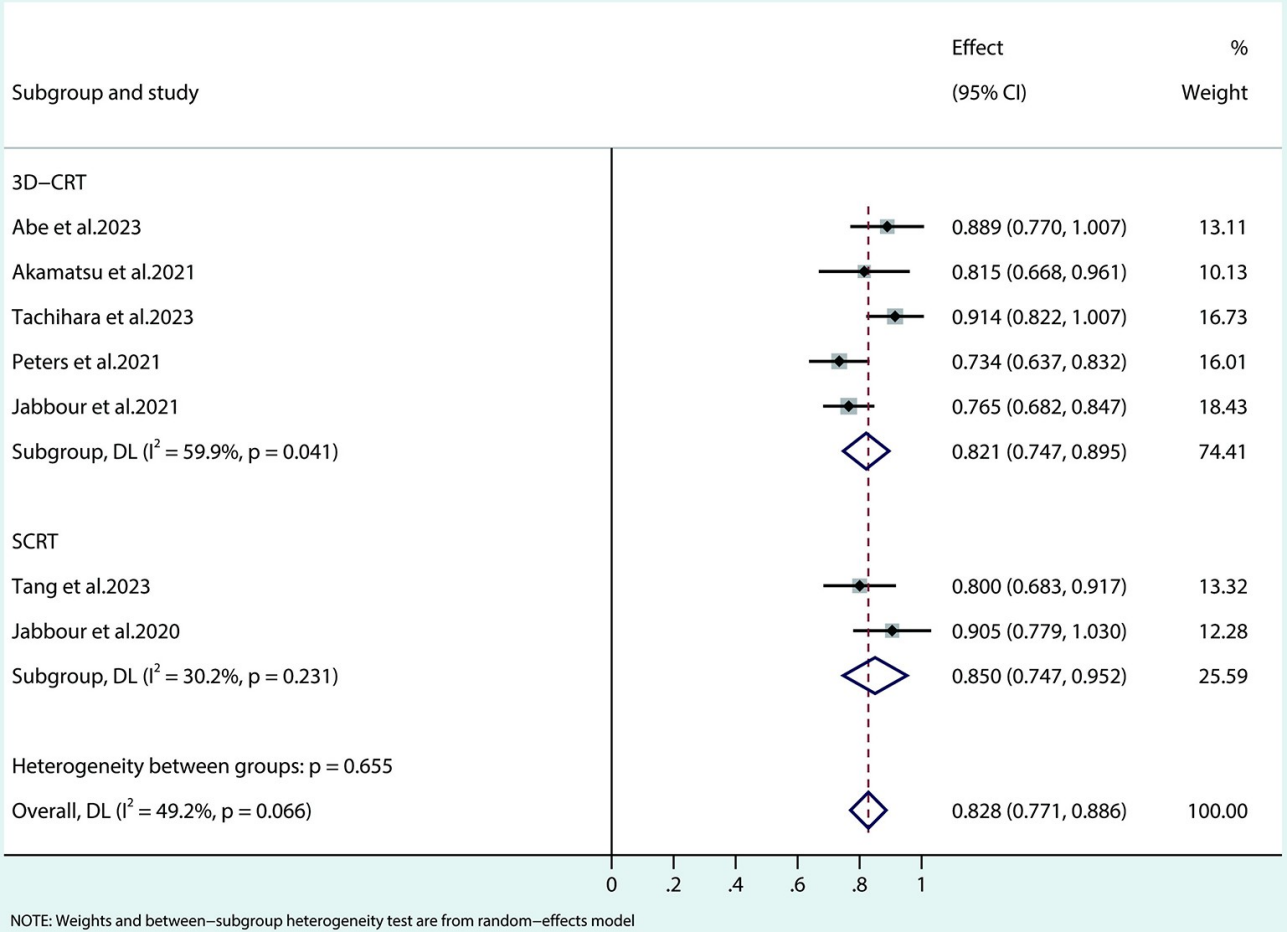
Forest plot of the pooled ORR based on various radiotherapy modalities.

The meta-analysis of ORR (Fig 4) showed that in the 3D-CRT subgroup, individual study effect sizes ranged from 0.734 to 0.914, with a pooled estimate of 0.821 (95% CI: 0.747, 0.895), indicating a significant tumor reduction in 82.1% of patients. However, moderate subgroup heterogeneity (I^2^ = 59.9%, p = 0.041) suggests potential variability in ORR outcomes, possibly due to differences in study design, patient characteristics, or treatment protocols. In contrast, the SCRT subgroup demonstrated individual study effect sizes of 0.800 and 0.905, with a pooled estimate of 0.850 (95% CI: 0.747, 0.952), implying an average response rate of 85.0%. The subgroup exhibited low heterogeneity (I^2^ = 30.2%, p = 0.231), indicating a higher level of consistency among the included studies. There was no statistically significant overall heterogeneity between the subgroups (p = 0.655), and the combined ORR for both subgroups was 0.828 (95% CI: 0.771, 0.886), demonstrating substantial efficacy for both modalities, albeit with a slight advantage for SCRT.

**Fig 5:**
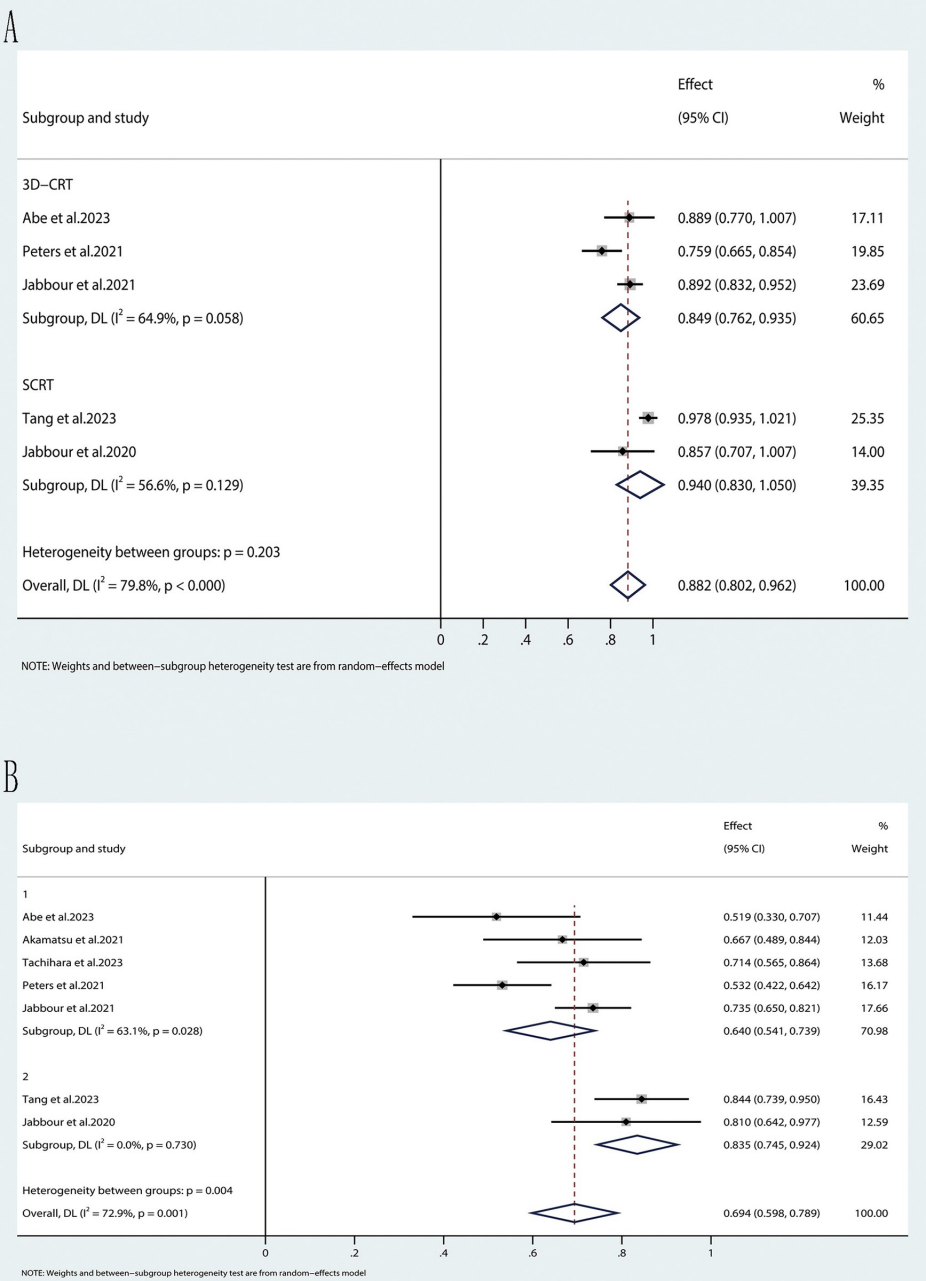
Forest plot of the pooled results for OS (A) and PFS (B) according to different radiotherapy modalities.

In the OS analysis (Fig 5A), three trials within the 3D-CRT subgroup presented effect estimates (hazard ratios) of 0.889, 0.759, and 0.892, with 95% confidence intervals excluding 1, suggesting potential statistical significance. The combined effect for this subgroup was 0.849 (95% CI: 0.762, 0.935). The SCRT subgroup, consisting of two studies, displayed effect estimates of 0.978 and 0.857, with 95% CIs encompassing 1, indicating no statistically significant disparities. The combined effect for the SCRT subgroup was 0.940 (95% CI: 0.830, 1.050). The heterogeneity between the subgroups was not significant (p = 0.203), and the overall combined effect was 0.882 (95% CI: 0.802, 0.962), with considerable overall heterogeneity (I^2^ = 79.8%).

The PFS analysis (Fig 5B) comprised four trials within the 3D-CRT subgroup, presenting effect estimates ranging from 0.519 to 0.714, with all 95% CIs excluding 1, indicating potential statistical significance. The collective effect for this subgroup was 0.640 (95% CI: 0.541, 0.739). In contrast, the SCRT subgroup, consisting of two studies, reported effect estimates of 0.844 and 0.810, with 95% CIs including 1, implying no statistically significant differences. The combined effect for the SCRT subgroup was 0.835 (95% CI: 0.745, 0.924). Significant heterogeneity between the subgroups was noted (p = 0.004), and the overall combined effect was 0.694 (95% CI: 0.598, 0.789), with substantial overall heterogeneity (I^2^ = 72.9%).

### Toxicities

The most frequent adverse events (AEs) of all grades associated with the combination of immune checkpoint inhibitors (ICIs) and radiotherapy or chemoradiotherapy in treating stage III advanced non-small cell lung cancer (NSCLC) were analyzed and are summarized in Table 4. The majority of patients reported grade 1–2 AEs, which were generally well-tolerated. The analysis identified the three most common AEs as anemia, fatigue, and nausea, with incidences of 51% (95% CI: 32.5%–69.7%), 46.6% (95% CI: 18.1%–75.1%), and 47.6% (95% CI: 10.2%–85.0%), respectively. Additionally, the rates of radiotherapy-related AEs, such as pneumonitis and radiation pneumonitis, were found to be 36.3% (95% CI: 15.2%–57.4%). Importantly, the occurrence of grade III or higher AEs was significantly lower, with rare cases exceeding 10%. Specifically, the most frequently observed grade III or higher AEs—namely anemia, fatigue, and diarrhea—showed incidences of only 5.3% (95% CI: 0.5%–10.1%), 2.1% (95% CI: 0.1%–4.1%), and 3.6% (95% CI: 0.5%–6.7%), respectively. AEs leading to mortality were recorded at a rate of 3% (95% CI: 0.9%–5.1%). However, studies focusing on stage IV NSCLC did not provide complete and valid data on AEs; thus, preventing a statistical analysis of potential AEs in patients at this stage (please refer to the Supplementary material for more details).

**Table 4:** Adverse events of the studies included in the meta-analysis.

AE	All grade		≥Grade III	
	ES,% (95CI)	I2,%	ES,% (95CI)	I2,%
Anemia	0.466	69.9	0.053	45.9
Decreased appetite	0.282	63.9	0.022	0
Diarrhea	0.195	43.7	0.036	0
Fatigue	0.51	95.3	0.021	0
Nausea	0.476	96.6	N/A	N/A
Neutrophil count decreased	0.307	84.1	0.09	0
Pneumonitis	0.286	97.4	0.05	47.5
Radiation pneumonitis	0.363	97.6	0.098	34.4
Rash	0.364	89.9	0.007	0
Vomiting	0.184	82.9	N/A	N/A

### Sensitivity analysis

The study conducted a sensitivity analysis by systematically excluding one study at a time to assess its impact on the combined results. The results of this analysis demonstrated that the pooled results, along with their 95% confidence intervals, remained relatively unchanged regardless of which study was excluded. This reaffirms the overall reliability of the meta-analysis results. Supplementary Figure provides a visual representation of the outcomes of the sensitivity analysis (please refer to the Supplementary material for more details).

### Publication bias

To ensure the robustness of the meta-analysis findings, Egger’s and Begg’s tests were employed to assess potential publication bias. The test results were largely consistent with the overall results. However, it is worth noting that there were indications of publication bias for diarrhea in terms of safety considerations (please refer to the Supplementary material for more details).

## Discussion

The efficacy of durvalumab following chemoradiotherapy in the treatment of [specific condition, e.g., non-small cell lung cancer] has been strongly supported by the PACIFIC trial, demonstrating both substantial and sustained benefits in OS and lasting improvements in PFS. At the five-year milestone, patients treated with durvalumab exhibited OS rates of 42.9% (95% confidence interval [CI]: 38.2%-47.4%), while the placebo group registered 33.4% (95% CI: 27.3%-39.6%). Similarly, the PFS rates for the durvalumab cohort reached 33.1% (95% CI: 28.0%-38.2%), significantly exceeding the 19.0% (95% CI: 13.6%-25.2%) observed in the placebo group. Furthermore, the stratified hazard ratio (HR) for OS was 0.72 (95% CI: 0.59%-0.89%), with a median OS of 47.5 months for the durvalumab group compared to 29.1 months for the placebo group. The stratified HR for PFS was equally noteworthy at 0.55 (95% CI: 0.45%-0.68%), and the median PFS was notably prolonged among durvalumab-treated patients at 16.9 months, in contrast to 5.6 months in the placebo cohort. These findings underscore durvalumab’s potential as a significant therapeutic advancement post-chemoradiotherapy. Subsequently, several authors have reported real-life data on the treatment of advanced NSCLC with ICIs after CRT, highlighting the combination of ICIs with radiotherapy or chemoradiotherapy as one of the most effective therapy strategies for advanced NSCLC [30–32]. A comprehensive meta-analysis conducted by Zhang et al., comprising 23 studies, including a cumulative patient cohort exceeding 4,400 individuals, has been published. Drawing from real-world data, this meta-analysis demonstrates that the short-term efficacy and safety profile of durvalumab align with the findings reported in the PACIFIC trial. The consistency of these results reinforces durvalumab’s application as a means to enhance clinical outcomes for patients with unresectable stage III NSCLC [10]. However, it is crucial to note that these investigations explicitly excluded patients experiencing disease progression before commencing ICIs following radiotherapy and chemotherapy. Notably, this subgroup constitutes approximately 20% to 30% of the clinical population. Addressing the management of this significant and underserved patient demographic is pressing, as it presents a critical gap in current therapeutic protocols. Elucidating optimal treatment strategies for these patients is essential for advancing the standard of care and improving outcomes for all patients affected by this condition. Recent research efforts have focused on exploring the efficacy and safety of concurrent ICIs with radiotherapy or chemoradiotherapy in advanced NSCLC, potentially improving the survival rate for those who missed the opportunity to receive ICIs after cCRT [33–35]. Here, our study provides a systematic review and meta-analysis of available data concerning the efficacy and safety of concurrent ICIs with radiotherapy or chemoradiotherapy in advanced NSCLC.

Our comprehensive systematic review and meta-analysis contribute significantly to the body of knowledge regarding the concurrent use of ICIs with radiotherapy or chemoradiotherapy for advanced non-small cell lung cancer (NSCLC). A notable aspect of our study is the incorporation of a critical yet frequently overlooked patient subset, comprising 20% to 30% of the clinical population, who undergo disease progression following radiotherapy and chemotherapy. By including this subgroup in our analysis, we provide a more comprehensive evaluation of therapeutic effectiveness and safety; thus, furnishing a robust evidentiary foundation to guide and enhance clinical decision-making within the broader NSCLC treatment context.

This meta-analysis elucidates the efficacy outcomes of concurrent ICIs with radiotherapy or chemoradiotherapy as a compelling treatment strategy, as reflected in the ORR, one-year OS rate, and one-year PFS rate. These findings address a gap in prior research, which had not definitively determined the complete potential of this approach. Specifically, the efficacy of concurrent ICIs with radiotherapy or chemoradiotherapy in advanced NSCLC demonstrated a wide range, with ORR spanning from 30% to 90.9%, including both stage III and IV NSCLC.

Subgroup analyses revealed a noteworthy ORR of 81.8% (95% confidence interval [CI]: 74.7%–88.9%) for stage III NSCLC, while stage IV NSCLC exhibited an ORR of 39.9% (95% CI: 27.4%–52.3%). Furthermore, the one-year PFS rate for stage III NSCLC stood at 68.2% (95% CI: 58.7%–77.7%), in contrast to 27.9% (95% CI: 20.8%–35.0%) for stage IV NSCLC. Patients with stage III NSCLC achieved a one-year OS rate of 86.2% (95% CI: 76.4%–96.0%), while those with stage IV disease experienced a one-year OS rate of 72.2% (95% CI: 53.7%–90.7%).

When compared to outcomes from studies employing ICIs after concurrent chemoradiotherapy, such as the PACIFIC trial, the one-year OS was reported at 83.1% (95% CI: 79.4%–86.2%), accompanied by a one-year PFS of 55.3% (95% CI: 50.5%–59.8%). A recent real-world meta-analysis of durvalumab following chemoradiation for unresectable stage III NSCLC corroborated these findings, reporting one-year PFS and OS rates of 65.5% (95% CI: 57.6%–74.4%) and 87.9% (95% CI: 82.6%–93.9%), respectively [33]. Furthermore, in a double-blind, phase III clinical study involving 616 patients with stage IV NSCLC, the reported ORR was 48.6%, with a one-year PFS rate of 34.1%, and a one-year OS rate of 61.7% [36].

The findings indicate that concurrent use of immune checkpoint inhibitors (ICIs) with radiotherapy or chemoradiotherapy may offer benefits similar to the traditionally studied sequential approach. This combination strategy holds promise, especially for patients at risk of disease progression during or after radiotherapy and chemotherapy. However, concerns persist regarding increased side effects with the combined use of radiotherapy and immunotherapy, given its relatively recent introduction as a treatment strategy [37]. Balancing efficacy and safety is crucial when combining ICIs with radiotherapy or chemoradiotherapy. Concurrent use of these therapies may lead to systemic effects, particularly an elevated risk of immune-related pneumonitis, which is the most severe reported adverse outcome. Our meta-analysis underscores the safety profile of concurrent ICIs with radiotherapy or chemoradiotherapy in advanced NSCLC. Although the incidence of grade 1–2 adverse events increased, the occurrence of grade 3 or higher serious adverse events did not surpass that reported in the PACIFIC trial, with mortality attributable to grade 5 or above events standing at 3% and 4.4%, respectively. These findings suggest that concurrent ICIs with radiotherapy or chemoradiotherapy result in manageable adverse events without worsening the severity of immune-related adverse events (irAEs).

Pneumonitis and radiation pneumonitis are common adverse events associated with the administration of ICIs and concurrent chemoradiotherapy. The combined use of these treatments may lead to increased pulmonary toxicity compared to each treatment alone. However, the occurrence of grade 3 or higher pneumonitis was reported to be below 10%. It is noteworthy that the primary contribution to grade 3 or higher pneumonitis among all studies included in this meta-analysis originated from KEYNOTE-799 [29], which reported an incidence rate of 4.0% for grade 3 or higher pneumonitis, slightly higher than the 3.4% reported in the PACIFIC trial. This variance may be attributed to several factors inherent to the KEYNOTE-799 study design, such as the concurrent administration of pembrolizumab with chemoradiotherapy, contrasting with the sequential use of durvalumab after chemoradiotherapy in the PACIFIC trial. Additionally, differences in patient characteristics, treatment regimens, and follow-up duration could contribute to the observed disparities in pneumonitis incidence. Furthermore, the occurrence of grade 3 or higher pneumonitis observed in this study aligns with established toxicity profiles of concurrent chemoradiotherapy for stage III NSCLC, and the overall safety profile is consistent with the adverse events observed with ICIs followed by radiotherapy/chemoradiotherapy in first-line advanced NSCLC (Table 5).

**Table 5:** Safety profile comparison similar trials.

Study	Antonia et al. [11]	Avrillon et al. [39]	Preti et al. [40]	Ellison et al. [41]
Patients, n	473	576	110	37
Age, years, median	64	64	66.3	66.5
Treatment	Durvalumab after Chemoradiotherapy	Durvalumab after Chemoradiotherapy	Durvalumab after Chemoradiotherapy	Durvalumab after Chemoradiotherapy
Any grade all causality AEs	460 (96.8)	371 (64.4%)	76(64.4%)	30 (77%)
Serious Immune Related Pneumonitis	16 (3.4%)	46(7.9%)	20(18.8%)	1(3%)
AE leading to death	21 (4.4%)	7 (1.2%)	3(2.7%)	1(3%)

These findings emphasize the critical need for vigilant monitoring and proactive management of irAEs when utilizing concurrent ICIs with radiotherapy or chemoradiotherapy. Future research efforts should prioritize the identification of predictive biomarkers and prognostic factors to stratify patients according to their susceptibility to developing severe adverse events, notably immune-related pneumonitis [38]. Through refined patient selection and optimized treatment approaches, the risk of life-threatening complications can be mitigated, leading to potential improvements in overall treatment outcomes.

Innovative approaches to reduce the occurrence and severity of immune-related pneumonitis should be investigated. Possible strategies could involve dose adjustments, altering the timing of ICIs and radiotherapy, or implementing prophylactic medications. By proactively addressing safety concerns linked to concurrent therapy, we can maximize the benefits of this promising treatment approach while safeguarding patient well-being.

Our meta-analysis sought to investigate the effect of concurrent immune checkpoint inhibitors combined with radiotherapy or chemoradiotherapy in advanced NSCLC. While the subgroup analysis comparing different radiotherapy modalities did not reveal statistically significant differences in this study, it is crucial to acknowledge the potential impact of radiotherapy dose, target volume, techniques, and immunotherapy approaches based on evidence from prior research.

The optimal radiotherapy dose and fractionation for maximizing synergistic effects with immunotherapy remain subjects of ongoing investigation. Preclinical studies suggest that hypofractionated radiotherapy, such as stereotactic body radiation therapy (SBRT), may augment immunomodulatory effects compared to conventional fractionation [42, 43]. Radiotherapy has the potential to trigger more potent anti-tumor immune responses by enhancing tumor antigen release, facilitating dendritic cell activation, and promoting cytotoxic T-cell infiltration [44]. Nevertheless, clinical evidence comparing different radiotherapy doses and fractionation alongside ICIs is limited. Additional prospective trials are warranted to ascertain the optimal radiotherapy regimen that optimizes systemic anti-tumor immunity.

The irradiated target volume is another critical factor that could impact the effectiveness of combining radiotherapy with ICIs. Although our study did not specifically investigate target volumes, emerging evidence suggests that irradiating larger tumor volumes or multiple metastatic sites might result in improved outcomes by enhancing the diversity of tumor-associated antigens and facilitating a more robust abscopal effect [45]. However, it is essential to carefully weigh the potential toxicity risks associated with larger irradiation volumes against the immunomodulatory advantages.

Furthermore, advancements in radiotherapy techniques, such as intensity-modulated radiotherapy (IMRT), image-guided radiotherapy (IGRT), and proton beam therapy (PBT), have facilitated more accurate tumor targeting while minimizing radiation exposure to normal tissues. These modern radiotherapy methods hold potential in reducing the incidence of radiation-induced toxicities when combined with ICIs, particularly in lowering the occurrence of radiation pneumonitis. Although our study did not pinpoint an optimal radiotherapy approach through subgroup analysis due to restricted sample sizes and the utilization of multiple radiotherapy techniques in some included trials, we anticipate that forthcoming prospective studies comparing advanced radiotherapy modalities with ICIs will illuminate the most effective radiotherapy strategy to optimize treatment outcomes. By carefully balancing efficacy and toxicity profiles, we envision that the ideal combination of radiotherapy and ICI will not only enhance treatment efficacy but also minimize adverse events, offering patients with advanced NSCLC safer and more efficient radiotherapy-immunotherapy options.

The selection of specific ICI agents and their sequencing with radiotherapy could also influence treatment outcomes. Our analysis encompassed studies employing various ICIs, including PD-1 inhibitors (nivolumab, pembrolizumab), PD-L1 inhibitors (durvalumab, atezolizumab), and CTLA-4 inhibitors (ipilimumab). Although all these agents enhance T-cell-mediated anti-tumor immunity, they target different pathways and may interact with radiotherapy differently [46, 47]. However, the optimal sequencing strategy remains contentious and necessitates further clinical validation.

When incorporating ICIs with radiotherapy or chemoradiotherapy, identifying predictive biomarkers is essential for optimizing patient selection and cost-effectiveness. For patients at low risk of progression, ICIs may offer marginal benefits at a high cost. Conversely, for high-risk patients, the potential survival benefits may outweigh the costs. Developing biomarker-based models that utilize PD-L1 expression, tumor mutational burden, and tumor-infiltrating lymphocytes is crucial for enhancing treatment precision. Ongoing research should focus on validating these biomarkers to refine patient stratification, thereby reducing unnecessary treatments and directing resources toward patients most likely to benefit. Further exploration of novel biomarkers and their interactions could enhance our understanding of the tumor immune environment and responsiveness to ICI.

However, some limitations are worth noting. First, the included studies were all noncontrolled trials with small sample sizes and some studies with low evidence; thus, we evaluated only the efficacy and adverse events without definite conclusions. Second, high heterogeneity existed among the included studies. We only performed a subgroup analysis of the different stages. Other factors, such as baseline characteristics, histological classification, mutation frequency, and different ICIs, could also result in heterogeneity. Thirdly, the reporting of baseline pulmonary function, smoking history, and performance status data was inconsistent and inadequate across the included studies, which impeded meaningful analyses of these factors. Fourth, due to the lack of sufficient data on patients with mutations, we were unable to analyze the effectiveness and safety of concurrent ICIs and chemoradiotherapy in these patients. However, further analysis is needed in the future to determine the optimal treatment. Fifth, most of the related research has not yet reached the endpoint, so there was no final analysis of survival data, and the credibility of the evidence was insufficient. Therefore, additional large-scale RCTs should be designed to systematically assess and report objective measures of baseline pulmonary function, detailed smoking history, and performance status, and to confirm the clinical effectiveness and safety of concurrent ICIs with radiotherapy or chemoradiotherapy in comparison to those of the currently adopted first-line treatment schemes.

## Conclusion

In summary, our meta-analysis establishes the effectiveness and safety of concurrent administration of ICIs with chemoradiotherapy in patients with advanced NSCLC, particularly for individuals in stage III who may undergo disease progression before the commencement of ICIs following radiotherapy and chemotherapy. These findings offer valuable insights and provide a foundation for considering phase III large-scale, multicenter RCTs in advanced NSCLC.

## Supporting information

S1 Checklist PRISMA 2020 checklist. (DOCX)

S1 File (RAR)

S2 File (RAR)

## References

[1] Cancer.Net Editorial Board. Lung cancer—non-small cell: statistics, 2023. Available from: https://www.cancer.net/cancer-types/lung-cancer-non-small-cell/statistics

[2] Mithoowani H, Febbraro M. Non-small-cell lung cancer in 2022: A review for general practitioners in oncology. Curr Oncol. 2022;29: 1828–39. doi: 10.3390/curroncol29030150 35323350 PMC8946954

[3] Jasper K, Stiles B, McDonald F, Palma DA. Practical management of oligometastatic non–small-cell lung cancer. J Clin Oncol. 2022;40: 635–41. doi: 10.1200/JCO.21.01719 34985915

[4] Aupérin A, Le Péchoux C, Rolland E, Curran WJ, Furuse K, Fournel P, et al. Meta-analysis of concomitant versus sequential radiochemotherapy in locally advanced non-small-cell lung cancer. J Clin Oncol. 2010;28: 2181–90. doi: 10.1200/JCO.2009.26.2543 20351327

[5] Herbst RS, Baas P, Kim DW, Felip E, Pérez-Gracia JL, Han JY, et al. Pembrolizumab versus docetaxel for previously treated, PD-L1-positive, advanced non-small-cell lung cancer (KEYNOTE-010): a randomised controlled trial. Lancet. 2016;387: 1540–50. doi: 10.1016/S0140-6736(15)01281-7 26712084

[6] Mok TSK, Wu YL, Kudaba I, Kowalski DM, Cho BC, Turna HZ, et al. Pembrolizumab versus chemotherapy for previously untreated, PD-L1-expressing, locally advanced or metastatic non-small-cell lung cancer (KEYNOTE-042): a randomised, open-label, controlled, phase 3 trial. Lancet. 2019;393: 1819–30. doi: 10.1016/S0140-6736(18)32409-7 30955977

[7] Ettinger DS, Wood DE, Akerley W, Bazhenova LA, Borghaei H, Camidge DR, et al. NCCN guidelines insights: non–small cell lung cancer, version 4.2016. J Natl Compr Canc Netw. 2016;14: 255–64. doi: 10.6004/jnccn.2016.0031 26957612 PMC10181272

[8] Spigel DR, Faivre-Finn C, Gray JE, Vicente D, Planchard D, Paz-Ares L, et al. Five-year survival outcomes from the PACIFIC trial: durvalumab after chemoradiotherapy in stage III non–small-cell lung cancer. J Clin Oncol. 2022;40: 1301–11. doi: 10.1200/JCO.21.01308 35108059 PMC9015199

[9] Wu J, Ni T, Deng R, Li Y, Zhong Q, Tang F, et al. Safety and efficacy of radiotherapy/chemoradiotherapy combined with immune checkpoint inhibitors for non-small cell lung cancer: A systematic review and meta-analysis. Front Immunol. 2023;14: 1065510. doi: 10.3389/fimmu.2023.1065510 36993952 PMC10040597

[10] Zhang Y, Tian Y, Zheng L, Sun X, Zhao Z, Zheng Y, et al. Efficacy and safety of consolidation durvalumab after chemoradiation therapy for stage III non-small-cell lung cancer: a systematic review, meta-analysis, and meta-regression of real-world studies. Front Pharmacol. 2023;14: 1103927. doi: 10.3389/fphar.2023.1103927 37361225 PMC10285075

[11] Antonia SJ, Villegas A, Daniel D, Vicente D, Murakami S, Hui R, et al. Durvalumab after chemoradiotherapy in stage III non–small-cell lung cancer. N Engl J Med. 2017;377: 1919–29. doi: 10.1056/NEJMoa1709937 28885881

[12] Ettinger DS, Wood DE, Aisner DL, Akerley W, Bauman JR, Bharat A, et al. NCCN guidelines® insights: Non-small cell lung cancer, version 2.2023. J Natl Compr Canc Netw. 2023;21: 340–50. doi: 10.6004/jnccn.2023.0020 37015337

[13] Xuan L, Bai C, Ju Z, Luo J, Guan H, Zhou PK, et al. Radiation-targeted immunotherapy: A new perspective in cancer radiotherapy. Cytokine Growth Factor Rev. 2023. doi: 10.1016/j.cytogfr.2023.11.003 38061920

[14] Brandmaier A, Formenti SC. The impact of radiation therapy on innate and adaptive tumor immunity. Semin Radiat Oncol. 2020;30: 139–44. doi: 10.1016/j.semradonc.2019.12.005 32381293

[15] Procureur A, Simonaggio A, Bibault JE, Oudard S, Vano YA. Enhance the immune checkpoint inhibitors efficacy with radiotherapy induced immunogenic cell death: A comprehensive review and latest developments. Cancers (Basel). 2021;13. doi: 10.3390/cancers13040678 33567530 PMC7915834

[16] Davar D, Zarour HM. Immunological targets for immunotherapy: inhibitory T cell receptors. Methods Mol Biol. 2020;2055: 23–60. doi: 10.1007/978-1-4939-9773-2_2 31502146 PMC7382898

[17] Du SS, Chen GW, Yang P, Chen YX, Hu Y, Zhao QQ, et al. Radiation therapy promotes hepatocellular carcinoma immune cloaking via PD-L1 upregulation induced by cGAS-STING activation. Int J Radiat Oncol Biol Phys. 2022;112: 1243–55. doi: 10.1016/j.ijrobp.2021.12.162 34986380

[18] Galluzzi L, Aryankalayil MJ, Coleman CN, Formenti SC. Emerging evidence for adapting radiotherapy to immunotherapy. Nat Rev Clin Oncol. 2023;20: 543–57. doi: 10.1038/s41571-023-00782-x 37280366

[19] Eisenhauer EA, Therasse P, Bogaerts J, Schwartz LH, Sargent D, Ford R, et al. New response evaluation criteria in solid tumours: revised RECIST guideline (version 1.1). Eur J Cancer. 2009;45: 228–47. doi: 10.1016/j.ejca.2008.10.026 19097774

[20] Abe T, Iino M, Saito S, Aoshika T, Ryuno Y, Ohta T, et al. Comparison of the efficacy and toxicity of concurrent chemoradiotherapy and durvalumab and concurrent chemoradiotherapy alone for locally advanced non-small cell lung cancer with N3 lymph node metastasis. Anticancer Res. 2023;43: 675–82. doi: 10.21873/anticanres.16205 36697072

[21] Akamatsu H, Murakami H, Harada H, Shimizu J, Hayashi H, Daga H, et al. Gefitinib with concurrent thoracic radiotherapy in unresectable locally advanced NSCLC with EGFR mutation; West Japan Oncology Group 6911L. J Thorac Oncol. 2021;16: 1745–52. doi: 10.1016/j.jtho.2021.05.019 34116229

[22] Bestvina CM, Pointer KB, Karrison T, Al-Hallaq H, Hoffman PC, Jelinek MJ, et al. A phase 1 trial of concurrent or sequential ipilimumab, nivolumab, and stereotactic body radiotherapy in patients with stage IV NSCLC study. J Thorac Oncol. 2022;17: 130–40. doi: 10.1016/j.jtho.2021.08.019 34500113

[23] Liu Y, Yao L, Kalhor N, Carter BW, Altan M, Blumenschein G, et al. Final efficacy outcomes of atezolizumab with chemoradiation for unresectable NSCLC: The phase II DETERRED trial. Lung Cancer. 2022;174: 112–7. doi: 10.1016/j.lungcan.2022.10.006 36371941

[24] Welsh J, Menon H, Chen D, Verma V, Tang C, Altan M, et al. Pembrolizumab with or without radiation therapy for metastatic non-small cell lung cancer: a randomized phase I/II trial. J Immunother Cancer. 2020;8. doi: 10.1136/jitc-2020-001001 33051340 PMC7555111

[25] Tachihara M, Tsujino K, Ishihara T, Hayashi H, Sato Y, Kurata T, et al. Durvalumab plus concurrent radiotherapy for treatment of locally advanced non-small cell lung cancer: The DOLPHIN phase 2 nonrandomized controlled trial. JAMA Oncol. 2023;9: 1505–13. doi: 10.1001/jamaoncol.2023.3309 37676681 PMC10485744

[26] Jabbour SK, Berman AT, Decker RH, Lin Y, Feigenberg SJ, Gettinger SN, et al. Phase 1 trial of Pembrolizumab administered concurrently with Chemoradiotherapy for locally advanced non–small cell lung cancer: a nonrandomized controlled trial. JAMA Oncol. 2020;6: 848–55. doi: 10.1001/jamaoncol.2019.6731 32077891 PMC7042914

[27] Peters S, Felip E, Dafni U, Tufman A, Guckenberger M, Álvarez R, et al. Progression-free and overall survival for concurrent nivolumab with standard concurrent chemoradiotherapy in locally advanced stage IIIA-B NSCLC: results from the European Thoracic Oncology Platform NICOLAS Phase II Trial (European Thoracic Oncology Platform 6–14). J Thorac Oncol. 2021;16: 278–88. doi: 10.1016/j.jtho.2020.10.129 33188912

[28] Tang S, Cong X, Zheng D, Chen C, Liu Z, Gao J, et al. Concurrent sintilimab with sequential chemoradiotherapy for unresectable, stage III non-small cell lung cancer: a retrospective study. Front Oncol. 2023;13: 1129989. doi: 10.3389/fonc.2023.1129989 37152047 PMC10157220

[29] Jabbour SK, Lee KH, Frost N, Breder V, Kowalski DM, Pollock T, et al. Pembrolizumab plus concurrent chemoradiation therapy in patients with unresectable, locally advanced, stage III non–small cell lung cancer: The phase 2 KEYNOTE-799 nonrandomized trial. JAMA Oncol. 2021;7: 1–9. doi: 10.1001/jamaoncol.2021.2301 34086039 PMC8446818

[30] Denault MH, Feng J, Kuang S, Shokoohi A, Leung B, Liu M, et al. Beyond PACIFIC: Real-world outcomes of adjuvant durvalumab according to treatment received and PD-L1 expression. Curr Oncol. 2023;30: 7499–507. doi: 10.3390/curroncol30080543 37623024 PMC10453050

[31] Bruni A, Scotti V, Borghetti P, Vagge S, Cozzi S, D’Angelo E, et al. A real-world, multicenter, observational retrospective study of durvalumab after concomitant or sequential chemoradiation for unresectable stage III non-small cell lung cancer. Front Oncol. 2021;11: 744956. doi: 10.3389/fonc.2021.744956 34650927 PMC8507147

[32] Desilets A, Blanc-Durand F, Lau S, Hakozaki T, Kitadai R, Malo J, et al. Durvalumab therapy following chemoradiation compared with a historical cohort treated with chemoradiation alone in patients with stage III non-small cell lung cancer: A real-world multicentre study. Eur J Cancer. 2021;142: 83–91. doi: 10.1016/j.ejca.2020.10.008 33242835

[33] Conibear J. Rationale for concurrent chemoradiotherapy for patients with stage III non-small-cell lung cancer. Br J Cancer. 2020;123: 10–7. doi: 10.1038/s41416-020-01070-6 33293671 PMC7735212

[34] Guan S, Ren K, Zhang X, Yan M, Li X, Zhao L. Concurrent chemoradiotherapy versus radiotherapy alone after induction chemoimmunotherapy for stage III NSCLC patients who did not undergo surgery: a single institution retrospective study. Radiat Oncol. 2023;18: 122. doi: 10.1186/s13014-023-02305-5 37491257 PMC10367242

[35] Kashihara T, Nakayama Y, Okuma K, Takahashi A, Kaneda T, Katagiri M, et al. Impact of interstitial lung abnormality on survival after adjuvant durvalumab with chemoradiotherapy for locally advanced non-small cell lung cancer. Radiother Oncol. 2023;180: 109454. doi: 10.1016/j.radonc.2022.109454 36640944

[36] Gandhi L, Rodríguez-Abreu D, Gadgeel S, Esteban E, Felip E, De Angelis F, et al. Pembrolizumab plus chemotherapy in metastatic non–small-cell lung cancer. N Engl J Med. 2018;378: 2078–92. doi: 10.1056/NEJMoa1801005 29658856

[37] Wu L, Zhang Z, Bai M, Yan Y, Yu J, Xu Y. Radiation combined with immune checkpoint inhibitors for unresectable locally advanced non-small cell lung cancer: Synergistic mechanisms, current state, challenges, and orientations. Cell Commun Signal. 2023;21: 119. doi: 10.1186/s12964-023-01139-8 37221584 PMC10207766

[38] Guberina N, Wirsdörfer F, Stuschke M, Jendrossek V. Combined radiation- and immune checkpoint-inhibitor-induced pneumonitis—The challenge to predict and detect overlapping immune-related adverse effects from evolving laboratory biomarkers and clinical imaging. Neoplasia. 2023;39: 100892. doi: 10.1016/j.neo.2023.100892 37011458 PMC10124136

[39] Avrillon V, Daniel C, Boisselier P, Le Péchoux C, Chouaid C. Nationwide real-life safety and treatment exposure data on durvalumab after concurrent chemoradiotherapy in unresectable stage iii, locally advanced, non-small cell lung cancer: analysis of patients enrolled in the french early access program. Lung. 2022;200: 95–105. doi: 10.1007/s00408-022-00511-8 35141799

[40] Preti BTB, Sanatani MS, Breadner D, Lakkunarajah S, Scott C, Esmonde-White C, et al. Real-world analysis of durvalumab after chemoradiation in stage III non-small-cell lung cancer. Curr Oncol. 2023;30: 7713–21. doi: 10.3390/curroncol30080559 37623040 PMC10453685

[41] Ellison C, Martens M, Argote JA, Benz S, Currey A, Johnstone C, et al. High-grade pneumonitis events in unresectable, locally advanced non-small cell lung cancer patients treated with definitive chemoradiation followed by adjuvant durvalumab. JTO Clin Res Rep. 2023: 100537. doi: 10.1016/j.jtocrr.2023.100537 PMC11567112 39555223

[42] Chen Y, Gao M, Huang Z, Yu J, Meng X. SBRT combined with PD-1/PD-L1 inhibitors in NSCLC treatment: a focus on the mechanisms, advances, and future challenges. J Hematol Oncol. 2020;13: 105. doi: 10.1186/s13045-020-00940-z 32723363 PMC7390199

[43] Zayed S, Louie AV, Breadner DA, Palma DA, Correa RJM. Radiation and immune checkpoint inhibitors in the treatment of oligometastatic non-small-cell lung cancer: a practical review of rationale, recent data, and research questions. Ther Adv Med Oncol. 2023;15: 17588359231183668. doi: 10.1177/17588359231183668 37435562 PMC10331344

[44] Weichselbaum RR, Liang H, Deng L, Fu YX. Radiotherapy and immunotherapy: a beneficial liaison? Nat Rev Clin Oncol. 2017;14: 365–79. doi: 10.1038/nrclinonc.2016.211 28094262

[45] Janopaul-Naylor JR, Shen Y, Qian DC, Buchwald ZS. The abscopal effect: A review of pre-clinical and clinical advances. Int J Mol Sci. 2021;22. doi: 10.3390/ijms222011061 34681719 PMC8537037

[46] Dovedi SJ, Adlard AL, Lipowska-Bhalla G, McKenna C, Jones S, Cheadle EJ, et al. Acquired resistance to fractionated radiotherapy can be overcome by concurrent PD-L1 blockade. Cancer Res. 2014;74: 5458–68. doi: 10.1158/0008-5472.CAN-14-1258 25274032

[47] Twyman-Saint Victor C, Rech AJ, Maity A, Rengan R, Pauken KE, Stelekati E, et al. Radiation and dual checkpoint blockade activate non-redundant immune mechanisms in cancer. Nature. 2015;520: 373–7. doi: 10.1038/nature14292 25754329 PMC4401634

